# Tropical Beef: Is There an Axiomatic Basis to Define the Concept?

**DOI:** 10.3390/foods10051025

**Published:** 2021-05-09

**Authors:** Maria Salud Rubio Lozano, Tania M. Ngapo, Nelson Huerta-Leidenz

**Affiliations:** 1 Meat Science Laboratory, Centro de Enseñanza Práctica e Investigación en Producción y Salud Animal, Facultad de Medicina Veterinaria y Zootecnia, Universidad Nacional Autónoma de México, Cruz Blanca 486, San Miguel Topilejo, Mexico D.F. 14500, Mexico; msalud65@gmail.com; 2 Saint-Hyacinthe Research and Development Centre, Agriculture and Agri-Food Canada, 3600 Boulevard Casavant Ouest, Saint Hyacinthe, QC J2S 8E3, Canada; tania.ngapo@canada.ca; 3 Department of Animal & Food Sciences, Texas Tech University, Lubbock, TX 79409-2141, USA

**Keywords:** tropical, beef, meat quality, nutrient, composition

## Abstract

Cattle production in tropical regions has been estimated to account for just over half of cattle worldwide, yet it has not been demonstrated that sufficient similarities in the cattle exist to describe tropical cattle and, even less so, to characterize the meat from these animals. The aim of this review is to investigate the quality and nutrient composition of meat from cattle raised in the Tropics to determine if there is an axiomatic basis that would allow the definition of a concept of “tropical beef”. Tropical beef is the meat obtained from cattle raised in tropical environments, the population of which remains largely uncharacterized. Production systems in the Tropics are highly diverse but converge on the use of indigenous and *Bos indicus* breeds or *Bos indicus*-influenced crossbreeds under pasture feeding regimes. While some systems allow cattle to be slaughtered at ≤2 years of age, most often animals are ≥3 years. These production systems generally produce lean, low-yielding carcasses and tough (>46 N), lean (≤3.6% intramuscular fat) meat with a macronutrient composition otherwise similar to beef from animals raised elsewhere (72–74% moisture and 20–24% protein). Fatty acid profiles depend on the breed and production systems, while mineral content is influenced by the environment. Although lean and tough, tropical beef is highly acceptable to the consumers it serves, is culturally and traditionally relevant and, in many countries, contributes to food security. Consolidating the findings from animal and meat science studies in the Tropics has allowed the demonstration of an axiomatic basis defining “tropical beef” as a concept.

## 1. Introduction

It has been estimated that cattle production in tropical regions accounts for just over half of the cattle worldwide, equating to greater than 805 M head [1]. For such a significant source of beef, the volume of meat-related scientific literature actually undertaken in the Tropics is relatively modest, with most works focused primarily on animal production. Some of this animal science literature uses the terms “tropical beef” or “tropical cattle” to describe cattle raised in and/or adapted to tropical environments [2,3,4,5,6,7]. However, given that there is much geographic, cultural and economic variation in these environments, it is not surprising that this research varies widely in all aspects of animal production. Regardless, the undefined global terms of “tropical beef” and “tropical cattle” are often cited as descriptors in distinct studies. Yet, while there are commonalities among studies, it has not been demonstrated that sufficient similarities in the cattle exist to describe tropical cattle and, even less so, to characterize the meat from these animals.

The Tropics are the region of Earth surrounding the equator delimited at ±23.5 degrees in latitude by the Tropic of Cancer to the north and the Tropic of Capricorn to the south. The region constitutes 36% of the Earth’s landmass and includes more than 130 countries from Africa, America, Asia and Oceania, either wholly or partially [8,9]. According to the Köppen classification, there are three categories of tropical climates based on rainfall dynamics and an average annual temperature always above 18 °C: (a) wet equatorial climate (rainforest), (b) tropical monsoon and trade–wind littoral climate (monsoon), and (c) tropical wet–dry climate (savannah) [10,11].

By 2050, global meat consumption is expected to increase by 30% and at least 70% of the increase in beef production required to meet the growing demand is expected to come from the tropical and subtropical regions of the world [12]. Unless there are major changes in production systems, environmental conditions will always determine the types of livestock that can be used in the harsh tropical regions, even though these types may not necessarily meet the growing demand for meat and milk [13]. However, it is not only the climate that dictates animal production in many of these countries. In 2020, the United Nations estimated that some 43% of the world’s population, almost 3.8 billion people, live in the Tropics [9]. Of these, about 99% live in a nation considered to be “developing”, which includes 85% of the poorest people in the world. People living in the Tropics are also far more culturally diverse than in the rest of the world, exemplified by the fact that these regions account for more than 80% of all living languages [14].

These climatic, cultural and economic conditions have driven production systems in the Tropics to concord in the use of breeds (generally, *Bos indicus* and *Bos indicus* crosses), management systems (extensive and semi-intensive) and feed (pastures and finishing grain) and, consequently, produce carcasses of similar quality [15,16]. However, although similar, each region represents an important source of variation to provide meat that is acceptable to the consumer and is culturally and traditionally relevant [17]. These animals are often dual- (milk and meat) or multi-purpose (milk, beef, draught, fuel and fertilizer) and have important functions ranging from the provision of food and income to socioeconomic, cultural and ecological roles of farming communities [18,19]. Tropical cattle production systems make an important contribution to household food security and income for smallholder beef production. However, the majority of the tropical cattle populations remain largely uncharacterized, and the meat quality of these populations is even less explored.

The aim of this review is to investigate the quality and composition of meat from cattle raised in the Tropics to determine if there is an axiomatic basis that would allow the definition of the concept of “tropical beef”, where beef refers to the meat and not the animal. Literature cited in the present review was gathered through a range of databases, including, but not exclusively, Scopus, Food Science and Technology abstracts, Agricola, Biological abstracts, CAB abstracts and OVID medline, as well as extended library and online searches for texts on cattle raised in the Tropics. Keywords used included, but were not limited to, variations of Tropics, beef, cattle, breeds, *Bos indicus*, zebu, sanga, Criollo, meat, quality, nutrition, nutrient, composition, carcass, fatty acids, intramuscular fat, minerals, eating quality, tenderness, feedlot, pasture and production systems. The references from the articles obtained by this method were used to identify additional relevant material.

## 2. Cattle Production in the Tropics

It is inherent that, in order to define a concept of “tropical beef”, characterization of the cattle from which the meat is derived is first required. Reviewing similarities and differences between cattle production systems in the Tropics allows a description of tropical cattle and provides context in defining the resulting beef, given that almost all aspects of animal production impact meat quality and nutrient composition to some degree. The importance of a holistic approach to understanding tropical beef quality and composition is exemplified in the description of the strong growth of Brazilian cattle production described by Ferraz and de Felício [20] as being based on a triploid of Nellore-cerrado-*Brachiaria* grass (that is, breed-environment and production system-feed) since 1970. A large body of scientific work reports on genetics and production of cattle in tropical environments. It is beyond the scope of this study to review these aspects, but rather, the focus of this section is to provide context in order to define and understand the characteristics of the meat obtained from “tropical cattle”.

### 2.1. Breeds

The cattle breeds of preference for production in tropical climates are generally *Bos indicus* or *Bos indicus* crossbreeds. Indeed, Meat and Livestock Australia [21] actually describes *Bos indicus* as tropical breed cattle genetically adapted to survive and produce under adverse conditions, including heat and poor-quality pastures. Unique evolutionary traits of *Bos indicus* breeds, also known as zebu, to tropical climates are well-documented and include resistance to some ecto- and endoparasites and endemic diseases, heat and drought tolerance and other harsh environmental conditions, such as limited water, poor pasture and high humidity [16,22,23,24,25,26,27,28,29,30]. Although adapted to the local environments, *Bos indicus* cattle are often poor milk and meat producers [31]. Furthermore, poor production performance traits, carcass conformation, and meat marbling content and eating quality are also generally associated with these breeds [16,20,24,32,33,34,35]. Consequently, crosses with *Bos taurus* breeds are much studied, given that crossbreeding represents a proven strategy to improve the adaptation almost immediately. Indeed, heterosis has been demonstrated to influence cattle body temperature maintenance, reproduction, survival and, to a lesser extent, temperament in subtropical or stressful environmental conditions, such as toxic fescue [36,37,38]. However, while crossbreeding might improve the carcass, meat and sensory quality traits, generally, the higher the proportion of *Bos taurus*, the lesser the adaptability to the tropical environment [33,39].

The most commonly used *Bos indicus* breeds in research appear to be Nellore and Brahman, likely a result of the use of these breeds in large-scale commercial meat chains. Indeed, studies on Nellore are predominantly from the research undertaken in Brazil, the country with the world’s largest commercial herd, of which the Brazilian Zebu Breeders Association claim that 80% has influence from zebu cattle, and the breed with the largest number of animals is the Nellore [20]. However, there are other breeds of significant number in Brazil, including Guzerat, Gyr, Indubrasil and Tabapua, and interest has also been shown in *Bos taurus* breeds adapted to tropical environments, such as Brazilian Caracu, as well as the introduction of breeds like Senepol and Bonsmara and composite programs, such as Montana Tropical [20]. Crossbreeds and composites are prevalent throughout cattle production, and research reports on tropical herd improvement by crossbreeding date back over a century. Some crosses, such as the Senepol and Bonsmara, have even been developed to recognition as breeds in their own right, including, for example, Brangus, Santa Gertrudis and Charbray [21]. In many countries, artificial selection and management interventions have resulted in marked productivity improvements and by extension, economic performance for commercial cattle breeds [40,41]. However, this is not universal, and for example, in Africa, the focus of selection has predominantly been on survival, in often unpredictable, harsh and changing environmental conditions, and not consistently for productivity gains [42].

In addition to the predominantly *Bos indicus* indigenous breeds and crosses found in tropical countries, in Latin America, there is a group of *Bos taurus* cattle referred to as Criollo. Criollo have the ability to adapt to harsh arid landscapes with minimal human intervention [43]. Some Criollo cattle have been developed into unique breeds, such as the tropically adapted Romosinuano of Colombia, while others are responsible for the genetics that led to the Texas Longhorn. While not common, some small, isolated populations of Criollo in Mexico have not been crossbred at all [43]. These cattle are not part of Mexico’s commercial market, due, in large part, to being light-muscled and having non-uniform conformation [43,44]. As for indigenous cattle, many of the characteristics and traits attributed to Criollo have yet to be verified scientifically.

While there is an overwhelming amount of research reporting on genetic selection and production of tropical breeds, these encompass but a few of the many breeds that are found in tropical climates, most of which are little described in the scientific literature, if at all. For example, a survey conducted as part of a large effort to systematically collate information aimed at assessing the status of the cattle genetic resources of sub-Saharan Africa describes 145 cattle breeds/strains little reported elsewhere [27,45]. The large number of indigenous cattle breeds would suggest that there is significant genetic diversity of cattle in many parts of the world, yet many cattle breeds face extinction [42]. However, artificial selection and management have often been achieved at the cost of reduced genetic diversity and, in some cases, fertility [40,41]. For example, to compensate for the relatively low production potential of indigenous cattle, crossbreeding with exotic breeds is commonly practiced in Africa, with minimal within breed selection for the indigenous breeds [42]. The end result is a continual erosion and loss of cattle diversity, including for adaptive traits. To give an indication of the scale of this loss of diversity, in 1999, it was reported that 32% of indigenous African cattle breeds were in danger of extinction [45].

To summarize, there are a vast number of breeds of cattle grown in tropical regions, of which the majority appear to be *Bos indicus* or *Bos indicus*-influenced. There are also some tropically adapted *Bos taurus* breeds and types, but these appear of little interest to large-scale commercial operations. A large body of scientific literature reports on the breeding and genetics of cattle in the Tropics, but until recently, the focus of this research has been primarily on improving production traits for financial gain. However, in parallel to the rapid evolution of genetic research tools, realities of climate change and ever-increasing erosion of diversity, so too has the focus of research evolved and nowadays encompasses carcass conformation, meat and sensory quality attributes, production traits that reduce the environmental footprint of production [44] and breed classification and description [27,45].

### 2.2. Production Systems

Beef cattle farming systems and supply chains vary according to geographical regions, availability of resources, infrastructure, urbanization and markets [46]. It is not surprising, therefore, that, in tropical countries, production systems run the gamut from large commercial operations specifically for meat production to farmers with but a few mixed-purpose cattle. As for breeding and the genetics of cattle in tropical climates, there is a vast amount of publications on animal production. Brief descriptions taken from select reviews serve to illustrate the diversity of the production systems in tropical climates.

In a review of Brazilian cattle production, Ferraz and de Felício [20] described that, at 305 M head of cattle, Brazil is second only to India (325 M head) in total cattle herd size and has the largest commercial cattle herd in the world. Cattle are raised on 1.8 million farms, ranging from small beef farms of less than 500 head per household per year to commercial operations with over 4000 head per year. While extensive production systems are the norm, an estimated 10% of Brazil’s meat production in 2019 was finished in feedlots as a means to limit the weight loss common in the dry season. To minimize the impact of the marked decrease in tropical forage quality and availability in the dry season, three production systems are employed [20]. The first is a complete pasture-based system in which controlled mating is used to start the calving season in November/December to February; calves are weaned May–June and kept on dryland pasture until the next rainy season in October. The animals lose weight during the dry season, and about half are slaughtered at 24–30 months, the balance at 36–42 months. In the second, finishers buy two-year-old steers and finish them in better pastures for one year. And, in the third system, calves are supplemented during pre-weaning to produce heavier weaned calves that go directly to one of three finishing schemes: (a) at 8 months and 240 kg live weight, animals (generally crossbreeds) are transferred to feedlots for 120 days and slaughtered at 420 kg, (b) weaned animals (pure and crossbreeds) are sent to pasture for a growing phase from 18–24 months, then transferred to feedlots, and (c) animals (mostly *Bos indicus* and some crossbred steers) are kept on pasture and slaughtered at 30–42 months and 450–500 kg. The average slaughter age for cattle in Brazil is 4 years [20].

In a more recent review of another Latin American country, Parra-Bracamonte, Lopez-Villalobos, Morris and Vázquez-Armijo [47] described cattle production systems in Mexico, which has around 31.7 M cattle. The five most-important beef production states in Mexico (Veracruz, Jalisco, San Luis Potosí, Tabasco and Chiapas) are in tropical and subtropical regions. Mexican beef originates from cow–calf production systems, which provide cattle for feeder or feedlot systems and for live export. Cow–calf operations in Mexico consist of purebred, multiplier and dual-purpose systems [47], the latter two systems being found in tropical regions. Nearly 90% of farms within these systems have more than 20 ha. Multiplier cow–calf herds are the most numerous and located in all agro-climatic regions. These are extensive pasture-based farms in which the main product is weaned calves. Dual-purpose cattle farming systems producing milk and meat comprise almost 9% of the cattle in Mexico [47]. These types of farms are located mostly in tropical regions. Meat produced for the domestic market is generally finished in feedlots, and all geographical regions have feedlot systems, but the levels of management and days of fattening vary with the region. In the tropical regions, longer periods at pasture and fewer days of fattening in feedlots are usual (for example, 70–90 days), compared to feedlots in the northern temperate, arid and semi-arid regions (for example, 130–150 days) [48].

Unlike Brazil and Mexico, Australia has only a very small proportion of wet tropics, and most of its beef production comes from a dry tropical environment, characterized by distinct wet and dry seasons [49]. Northern Australian grazing lands, including tropical regions, collectively support about 14 million head or 60% of the national beef herd [50]. The production systems are similar to those of Brazil, where millions of hectares are used for grazing, with few feedlots. Individual properties range from less than 1000 ha and fewer than 1000 head to over 1.5 M ha with more than 40,000 cattle. Traditionally, reducing the stocking rates to maximize the head performance on native tropical pastures has been the option of preference, slaughtering animals at 550–600 kg live weight at about 4 years of age [51]. Nowadays, feedlot or supplementary feeding strategies may be used to finish animals at a younger age and to improve the carcass and fat cover [20].

Indonesia provides a complete contrast to the above countries. In 2017, Agus and Widi [52] reported that the cattle population totaled about 16.6 M head. Of these, 90% are held by smallholder farming systems, with about 6.5 M farmers living in rural areas. The remaining 10% are from more commercial farmers (˂1% of all farmers) and large beef cattle companies. Smallholder farmers are those who keep between two and four head of cattle and use stall feeding in Java where the land is scarce to 50 head or more extensively grazed in other areas. The definition of small holder is a stark contrast to those in Australia and Brazil with 500–1000 head of cattle. While most other reviews have focused on the commercial production of cattle, Agus and Widi [52] noted the importance of livestock for smallholder livelihoods around the world. For poor households in Indonesia, as in many other tropical countries, the non-income benefits of keeping livestock are particularly important. These farmers keep cattle to produce meat for the urban market, to support cropping with manure, to provide draught power and as assets. These sentiments are also reiterated by Mwai, Hanotte, Kwon and Cho [42], who describe that, across the African continent, cattle remain major sociocultural assets, play important social–cultural roles in many African societies (such as, marriage and initiation), represent an important source of animal protein (dairy and beef), provide draught power and supply fertilizer through manure, which is also used as fuel by some communities. In Indonesia, both stall feeding and extensive systems use low-quality feed, mainly from crop residues as well as agricultural byproducts and other nonconventional feedstuffs, such as oil palm leaves, cassava foliage, cotton seed meal, seaweed and food wastes. In terms of feedlot operations, about 75% of cattle imported from Australia are destined for feedlot in Indonesia.

These selected reviews provide an overview of the enormous diversity of cattle production in tropical countries. Yet, there are similarities. In general, production is extensive and often on forage of relatively poor nutritional quality. Age at slaughter varies with animals achieving slaughter weight at ages greater than in temperate or sub-tropical climates, often around 4 years unless semi-intensive or intensive production systems are used. Nutrient and feed supplementation or introduction of legumes or specialized crops into pastures is recommended in some regions and particularly during dry seasons. Possibly as important as the introduction of *Bos taurus* genetics to improve carcass conformation, increase fat deposition and decrease age at slaughter, is the introduction of feedlots to tropical beef production. Although incipient, increasingly, cattle are finished in feedlots, particularly as a means to meet the demands of export markets. Alternative finishing options include the transfer to farms of higher quality forage and/or supplementation. However, all of these interventions are costly, and for many but large commercial operations, the cost may be prohibitive.

## 3. Carcass and Meat Quality

The most reported of carcass characteristics in research on tropical beef are the slaughter weight and dressing percentage, often included as an extension of cattle production studies. It is therefore not surprising that these characteristics are at the core of the scientific literature of beef carcass and meat quality research in tropical Africa, of which reports are relatively scarce. A study in Ghana found carcass weights of zebu cattle (156 kg average) heavier than sanga (93 kg), which were, in turn, heavier than West African Shorthorn (73 kg), these being from slaughter weights of 309, 201 and 162 kg, respectively [53]. In a review of Shorthorn cattle production in West and Central Africa, carcass weights ranged from 80 to 200 kg [54], and it was concluded that, owing to their small size, the performance of Shorthorn cattle was generally low. However, the dressing percentages (ranging from 42% to 55%) were similar to those of other breeds within and outside the region. In Uganda, it was also found that, while the indigenous genotypes are well-adapted to the tropical production environment, slow growth rates and smaller mature body weights limit their potential for meat production [55]. Here, beef production is described as evolving from traditional pastoral practices to sedentary semi-intensive systems on private ranches. Reflecting what is actually happening across the region, a study was undertaken comparing three locally available genotypes (pure Boran, Ankole x Holstein Freisian cross and a composite genotype) and finished either in pasture or in feedlots (60, 90 or 120 days) that use locally available agro-industrial byproducts. Bulls were 12 to 20 months old at slaughter and the average final live weights ranged from 198 to 238 kg on pasture compared to 221 to 279 kg in feedlots. Similarly, hot carcass weights were also higher for those animals fattened in feedlots (115–153 kg vs. 99–114 kg). Slaughter characteristics did not vary with genotype. In a Cameroonian study, breed also had only a limited effect on the carcass characteristics of cattle harvested in a local slaughterhouse [56]. In this study, 1953 carcasses from three local zebu breeds, Gudali, White Fulani and Red Mbororo, were evaluated, and body condition score, carcass weight and carcass conformation were highest in castrated males, while heifers had the highest fatness levels and bulls, the lowest. It was concluded that the month of year greatly influences the carcass weight, which increased from March to September and decreased from September to March. In an earlier study, an average loss in body weight of 13.3 kg/month was reported from December to April due to the poor quality of forage coinciding with the dry season (November to March) [57]. While breeds showed limited differences in carcass traits in these African studies, an impact on meat toughness was observed in a Cameroonian study [56]. Gudali meat was tougher (unaged shear force of 112 N) than White Fulani (72 N) and Red Mbororo (78 N). In a Beninese study, the tenderness of meat from Borgou, Lagunaire and Zebu Fulani cattle did not significantly differ, but did decrease from 91–122 N at slaughter to 37–66 N after 8 days aging [58]. In both of these studies, bulls were raised in pasture and selected at a local slaughterhouse at 3–5 years of age [56,58].

While only but a few studies from tropical Africa are reported, the challenges of achieving profitable slaughter weights in beef cattle production in pasture is a common research theme in studies of tropical beef production. When striving to meet markets where consumer demand for tenderness is a priority, meat from young animals is a prerequisite, exacerbating the need for increased live weight gains in tropical cattle. Indeed, Poppi, Quigley, da Silva and McLennan [49] illustrated that the target market determines the growth path, so that, for example, a targeted high slaughter weight (undefined) can be achieved at 3.5–4.5 years of age of the animal from extensive range land pastures in Australia with minimal inputs, providing a profitable production system in Northern Australia to meet the ground meat market in North America. However, this growth path cannot attain more profitable markets with exigent meat quality demands. This challenge has driven large-scale research programs in the region over the last 40 years.

Since the 1980s, in Australia, crosses of Brahman with other breeds have been investigated to improve the production and carcass characteristics in tropical regions. Ball and Johnson [59] found Brahman crossbred cattle to have higher saleable beef yield (1–3%) over Hereford cattle under tropical conditions. In a series of experiments, Wythes, Shorthose, Dodt and Dickinson [60] observed that slaughter and carcass weights, backfat thickness and shear force values of unaged meat were generally similar between steers of *Bos taurus* and crosses of *Bos indicus* × *Bos taurus*. In a follow-up study, chronological age and dentition had no significant impact on shear force values (76–99 N) of *M. longissimus dorsi* from cows and steers of Brahman and Brahman x Shorthorn or Hereford crossbreeds [61]. It was concluded that overall toughness of meat from cattle slaughtered in Northern Australia was of much greater concern than the minor differences between genotypes. Newman, Burrow, Shepherd and Bindon [62] noted that purebred Brahman had the highest peak shear force measurements (52–59 N, aging not specified) when compared to progeny of Brahman females mated to sires of eight different breeds (Brahman, Santa Gertrudis, Belmont Red, Angus, Hereford, Shorthorn, Charolais or Limousin). Furthermore, the average peak force values in Brahman cattle were considered above acceptable values for tenderness (no acceptability threshold was provided). In this study, it was also reported that European and British sire breeds produced consistently heavier carcasses than those from the progeny of tropically adapted breeds or Brahman sires [63]. When domestic market carcass weight (220 kg) was targeted, very small differences were found between sire breeds for carcass yield traits. However, when export market carcass weights (280 kg and 340 kg) were achieved, crossbreeds of Brahman and Charolais or Limousin produced leaner carcasses and greater yield percentages than other crossbreeds. In addition, differences in intramuscular fat (IMF) percentage among sire breeds were not observed at a 220 kg carcass weight (1.65%), but at 280 and 340 kg, increases in the IMF (2.28% and 2.85%, respectively) were consistent with increasing age [62]. It was concluded that the common practice of incurring fixed costs of slaughtering animals at lighter weights for the Australian domestic market to ensure a tender product is a fallacy and that considerable cost savings might accrue to processors and retailers who slaughter animals at heavier weights without any detrimental effects on meat tenderness.

In the same study, pasture and feedlot-finished steers and heifers were compared [62,63]. While much of the tropical beef research is focused on the use of crossbreeding to improve production and meat quality traits, over the last couple of decades, research on the use of feedlots, particularly for the finishing phase, has also come to the forefront. These workers found that animals finished at pasture were considerably older (739–805 days) and leaner (8.0–13.6 mm fat at P8 and 1.58–1.74% IMF) than those finished in feedlots (626–672 days, 11.5–15.8 mm fat at P8 and 2.09–2.30% IMF) and had larger eye muscle areas, higher retail beef yield percentages and the greatest weight of retail primals [62,63]. The meat from pasture-finished animals was also consistently tougher than that from feedlot-finished heifers (55 vs. 47 N, aging not specified).

In stark contrast to these earlier publications is a study reporting that Senepol × Brahman steers produced a more tender meat than purebred Brahman steers (shear force values after 14 days of aging of 34 N and 39 N, respectively) [64]. In addition, other than hump height, most of the carcass measures were similar for the two genotypes, and it was suggested that this crossbreed demonstrated a viable method to improve the meat quality of cattle produced in Northern Australia. These animals were raised in pasture and finished in feedlot. It was noted that all the meat from the purebred Brahman was relatively tender when compared to values that have been found for other Brahmans. The good tenderness results found for both genotypes in this study were considered likely due to the young slaughter age achieved (average estimated age of 21.5 month and hot carcass weight of 238 kg), and it was concluded that Brahman cattle with good meat quality can be produced by production systems that give good growth rates and minimize the age at slaughter. However, it was noted that this may not be possible on many extensive properties in northern Australia where growth rates are low, and it was cautioned that changing the growth path of Brahmans for slaughter at a younger age would not overcome grading penalties incurred as a consequence of a higher hump.

As for most of the carcass and meat quality research in tropical environments, in the Brazilian tropics, the use of crossbreeds is the primary focus of much of the published research. Norman and de Felicio [65] observed that, although some differences in the carcass composition of Nellore, Guzerat, Charolais and Canchim bulls could be attributed to breed effect, most were caused by the varying nutritional status of the animals pre-slaughter. Furthermore, lower hindquarter (45–46 kg vs. 47–48 kg) and higher forequarter (39 vs. 36–37 kg) weights were observed in the *Bos indicus* animals, attributed, at least in part, to earlier sexual maturity. Maggioni, Marques, Rotta, Perotto, Ducatti, Visentainer and do Prado [66] found that greater daily weight gains of bulls of crossbreeds (1/2 Nellore × 1/2 European or 1/4 Nellore × 3/4 European bulls) resulted in better carcass conformation (good vs. regular), thicker subcutaneous fat (3.38 vs. 1.92 mm) and a higher marbling score (light vs. trace) than those of purebred Nellore. Pflanzer and de Felicio [67] found that if slaughtering Nellore steers at a young age, the animals need to be fattened in order to achieve an acceptable marbling level. Bressan, Rodrigues, Rossato, Ramos and da Gama [68] found that meat from feedlot-finished animals was more tender than that from pasture-finished animals (55 vs. 59 N after 10 days of aging). However, these workers also found that *Bos taurus* cattle had lower shear force than *Bos indicus* (54 vs. 60 N), without reporting the actual breeds, other than to note that they were commercial bulls.

In a study of crosses of another extensively used breed in Brazil, Guzerat (Guzerat × Holstein, Guzerat × Nellore and 1/2 Simmental + 1/4 Guzerat + 1/4 Nellore), the three-cross had heavier cold carcass weights and greater rib-eye areas than the other crosses [69]. The crosses with Nellore were also tougher than that with Holstein (50.9 and 50.1 vs. 43.1 N shear force, respectively) [69]. Interestingly, one study compared a *Bos indicus* × *Bos indicus* crossbreed (Brahman × Nellore) with Angus × Nellore and purebred Nellore [70]. The carcass weights of both crossbreeds were heavier than those of purebred Nellore, and the proportion of carcasses grading Choice or Prime was greater in Angus × Nellore cattle than in the Brahman × Nellore or purebred Nellore cattle (26%, 12% and 16%, respectively). Steaks from Angus × Nellore calves were more tender than Nellore steaks, with the Brahman × Nellore steaks being intermediate (33, 42 and 39 N, respectively, after 14 days of aging). Significant variation among Nellore sires was observed for slaughter weight, dressing percentage, carcass weight, *longissimus* muscle area and marbling score, but not for backfat or shear force. The percentage of carcasses of Nellore cattle grading Choice or Prime ranged from 0% to 61.5%, and it was concluded that, while *Bos indicus* cattle have inferior carcass and meat quality relative to Angus × Nellore crossbreeds under tropical conditions, there is substantial variation within the Nellore breed for these traits, and several sires had a proportion of their progeny comparable in meat tenderness to those of Angus sires.

It should be noted that, in addition to shear force, a range of meat quality traits have been measured in studies of tropical beef, including ultimate pH, meat color, cooking and thawing losses, water-holding capacity, myofibrillar fragmentation index and sarcomere length, with few differences observed. Shear force is the exception, with a general consensus that *Bos indicus* breeds produce tough meat in tropical environments and tougher meat than *Bos taurus* breeds, often well-exceeding the minimum shear force of “very tough meat” (for example, 46 N [71], although it has also been reported as low as 38 N [72]), as is evident when compared in a tabulated form (Table 1). It is also apparent in the literature that many of the shear force measures are made without prior aging of the meat. If not aged, even a normally tender cut of beef can be expected to be tough. However, shear force measures at 1 to 2 days postmortem in many of these tropical countries reflect the local market in which beef aging is oftentimes rarely undertaken, such as in Venezuela [72], Costa Rica [73] and Mexico [74].

In Venezuela and Mexico, it is also reported that the occurrence of steers in the cattle population is atypical, as castration is rarely practiced [84,85]. In beef production, it is generally accepted that bulls provided adequate nutrition grow faster and more efficiently and produce carcasses with less fat than steers [86,87]. A higher proportion of cuts derived from the forequarter and a retained percent yield of total retail lean product at different weight ranges (from 163 to 365 kg carcass weight) has also been shown in bulls [88]. However, meat from steers is often preferred by consumers over meat from bulls because of the improved sensory traits, particularly tenderness [89,90]. In a Costa Rican study, it was reported that late castration (>12 months of age) had been reintroduced as a production tool to potentially increase the fatness and meat quality of subprimals of steers while taking advantage of the growth rates and efficiency of bulls [73]. However, few differences were observed in carcass and sub-primal yield traits of 3/4 Brahman ×1/4 Charolais bulls and steers raised on pasture and slaughtered at about 400kg live weight. *Longissimus lumborum* steaks from steers were more tender than those from bulls, whether castrated at 3, 7 or 12 months of age (100 vs. 86, 93 and 91 N, respectively), but *gluteus medius* was only significantly more tender from steers castrated at 3 months of age (64 vs. 73 N in bulls), and *semitendinosus* (about 62 N) and *psoas major* (about 39 N) were not different at any castration age. It was also observed that all but *psoas major* were significantly tougher at 2 days than 7, 14 and 28 days of aging, and for all four muscles, there were no significant differences between shear force values at 7 and 14 days of aging. Tenderness of the *longissimus lumborum* (76 N) and *gluteus medius* (57 N) was significantly improved at 28 days, but was still very tough in all except the *psoas major* (36 N; 60 N for *semitendinosus*).

In Mexico, young feedlot-finished bulls of six genotypes (zebu, European Brown Swiss, Holstein, zebu × European Brown Swiss, zebu × American Brown Swiss and zebu × Hereford) showed few significant differences in carcass and meat characteristics [78]. Of note was the higher shear force of the zebu (80 N) than all other genotypes (61–68 N). Another Mexican study similarly found that meat from feedlot-raised *Bos indicus* cattle was tougher than that from *Bos indicus* × *Bos taurus* crosses (73 vs. 55 and 59 N, respectively, after 14 days of aging), although all the samples were tough.

In a study of grading criteria of 23,484 beef carcasses in a commercial abattoir in a tropical region of Mexico, a beef carcass classification norm was implemented using five evaluation criteria applied in sequence: (1) maturity (age), (2) conformation (muscularity), (3) lean color, (4) fat color and (5) distribution of the subcutaneous fat cover [91]. The carcasses were classified as 13.4% Select, 45.8% Standard, 27.4% Commercial, 10.6% Out of Grade and 2.7% Veal, with no carcasses attaining the highest quality, Supreme grade. Based on maturity, 79.2% of the carcasses met the specifications for Supreme, but when the next criterion, conformation, was evaluated, only 0.5% of the carcasses graded Supreme. Using commercially purchased steaks, it was also found that beef from the central and southern regions of Mexico (regions where tropical production is prevalent) had greater shear force values than those from the northern (non-tropical) regions (46–47 vs. 36 N, respectively) [92]. Interestingly, while consumers also found beef from the north more tender than that from the other two regions, the overall desirability ratings were not significantly different. In addition, it has been observed that beef produced in the north of Mexico, which is largely based on feedlots, yields carcasses with a whiter fat than from the central and southern regions, where production relies more on pastures [84]. This finding corroborates other studies comparing feedlot and pasture-fed cattle [93,94], and with the emergence of feedlots, it is curious that the fat color is rarely, if ever, reported in the research on tropical beef.

In Venezuela, an analysis of carcass data from 590 bulls, steers and heifers showed that the dressing percentage of Zebu-type cattle outperformed the dairy/dual-purpose-type (64% vs. 54%), noting a wide range of values for the slaughter weights (285–657 kg), carcass weights (146–444 kg) and dressing percentages (47–71%) [95]. In the Venezuelan llanos, Brahman crossbreeds (× Romosinuano, Limousin, Angus, Gelbvieh or 3/4 *Bos taurus*) finished on pasture with supplementation achieved market weight (500 kg) with a desirable conformation at an earlier physiological age (shortened by 43 days) than those finished on pasture without supplementation [79]. The supplemented animals resulted in heavier (287 vs. 279 kg carcass weight) and fattier (1.26 vs. 0.88 cm backfat thickness) carcasses, but no differences were found for the low-yielding *longissimus* muscle area (79 cm^2^). Unexpectedly, supplementation produced meat with higher shear force values than pasture-only finishing (67 vs. 58 N), although all were very tough.

All of these studies illustrate that, while the limited carcass and meat quality research reports from tropical countries appear somewhat scattered and often use few animals, there are recurrent findings. Interventions are much-studied, and success is apparent in the use of crossbreeds, young bulls and feedlot finishing. The results from other studies, such as those of late castration and pasture finishing with supplementation, are not as promising, but the research is very limited to date. Regardless of the intervention, of which most are costly, tropical production systems generally result in low slaughter weights, lean carcasses and tough meat. Even with aging, crossbreeds, electrical stimulation and feedlot finishing, in general, tropical beef is very tough. In Brazil, Ferraz and de Felício [20] suggest a tender meat is achieved after aging during transport to export markets, but evidence is lacking in the scientific literature. Furthermore, this focus on the export market is indicative of much of the research on beef quality in tropical environments.

Research on tropical beef meat quality has been reported from a very limited number of countries—generally, those for whom export markets are of interest and research funding, resources and infrastructure follow as the sector strives to meet importers’ quality criteria. There is, therefore, a bias in the type of research undertaken targeting the quality criteria of non-tropical countries. Meat toughness may be a limiting factor for these more valuable export markets, but one can question the implementation of costly interventions for domestic markets. In many tropical countries, not only is the meat inherently tough, be it a consequence of tradition, food hygiene, lack of resources and infrastructure or for some other reason, meat is not aged. However, methods of food preparation often negate the necessity for a tender meat, and this is reflected in consumer perceptions. For example, it is reported that beef is rarely aged in Mexico [96], yet, in a survey of 488 Mexican consumers, 89% stated that the beef they buy is almost always or always of good quality, while only 1% reported that it is almost never or never good quality [97]. When asked how they prepare beef, the most popular methods were roasting, stewing and boiling (42%, 44% and 37%, respectively, noting that consumers could answer as many responses to this question as were appropriate). Only 26% said they fry beef and 6% grill. In the same survey, 59% of consumers preferred beef steaks with no marbling.

## 4. Nutrient Composition

Given the significance of marbling in export criteria as a meat quality indicator and the role of fat in the human diet, it is not surprising that there are a number of studies reporting the IMF content, generally with moisture and protein analyses, and fatty acid composition of beef from cattle raised in tropical environments. There are also a few studies of the mineral content, but no reports of amino acids or vitamins were apparent.

### 4.1. Macronutrients

Almost all the reports on meat from beef raised in the Tropics that include proximate analyses are from Latin America (Table 2). Compiled in tabulated form, it is readily apparent that there is generally little variation in the macronutrients of cattle grown in tropical regions. With the exception of three findings, the moisture ranges from 71% to 76%, and three quarters of the reported data are between 72% and 74%. The three excluded findings appear to be outliers, reporting very low moisture values [96,98]. Two of these data points are from a study comparing the use of anabolic steroid implants where low moisture contents (about 60%) and concomitant high protein contents (about 36%) were reported [96]. The third is from a study comparing meat from cattle raised on pasture with supplementation to feedlots, and the moisture contents of the latter were reported as 67.3%, likely, at least in part, a consequence of the high IMF content [98]. Indeed, in all of the studies reporting differences in moisture content, these data correspond with opposing differences in IMF content.

While almost all of the protein content values are in the range from 20% to 24%, some differences were observed between bulls and steers (22.9% vs. 23.8%, respectively [108]), breeds of Nellore and Nellore × European crosses (25.1% vs. about 23.8% respectively) [66] and pasture and grain finishing (21.4% vs. 18.4%, respectively [68]). However, these differences in the protein contents were relatively small, and the majority of the reports found no differences in the studies that covered a range of factors, including age at slaughter, fat class, carcass grade, sex, breed, feeding system and muscles.

The IMF content showed the greatest variation of the proximate analyses, ranging from 1.0% to 8.9%. However, the majority of the studies indicates a lean meat, with more than three-quarters of the data reporting an IMF of ≤3.6%. These findings are in accord with the low marbling and lean carcasses reported in tropical beef [62,63,66,67,84,92,94]. Given the importance of marbling score in the global marketplace, studies have been undertaken to better understand the low marbling scores in *Bos indicus*-influenced cattle compared to *Bos taurus* cattle [118,119,120]. In a review of these studies, it was noted that no strong relationship between the capacity to synthesize fatty acids *de novo* and the marbling score or adipocyte volume was reported, and it was concluded that the low marbling scores typically observed in *Bos indicus*-influenced cattle are mainly attributed to their smaller intramuscular adipocyte volume compared with *B. taurus* breeds [16]. Given that it is the volume of adipocytes, and not the quantity, that is of most consequence in explaining the difference in the genotypes, this reduced volume would also explain low IMF contents of tropical beef.

While generally low in cattle in tropical environments, there are reported differences of IMF contents with production characteristics. In three studies comparing slaughter ages, IMF content was observed higher in older animals. Cattle described as typical for Puerto Rico that were at least 3 years of age at slaughter had higher IMF than those of up to 2.5 years at slaughter (1.9% vs. 2.7%) [77]. In southern India, Kangayam bulls > 3 years of age had higher IMF contents than those 12–18 months old (2.89% vs. 2.09%) [110]. And, in Brazil, a progression of increasing fat contents with the age of Nellore steers was observed when grouped as 20–24 months (4.2%), 30–36 months (5.0%) and 42–48 months (5.7%) [67].

Differences in IMF content with sex are also reported in two Brazilian studies. One study found that pasture-finished Nellore × Aberdeen Angus steers had higher IMF content than bulls (1.96% and 0.95%) [108], while another reported that whole bulls had the lowest IMF content (1.23%), surgically castrated steers the highest (2.17%) and chemically castrated steers were intermediate and significantly different from both (1.61%) [107]. Increased accumulation of IMF through fat deposition induced by castration is primarily a result of an altered hormonal balance [121].

In terms of breed, the IMF content findings are inconsistent (Table 2). Lower IMF content was reported in Brahman than Charolais bulls (2.4% vs. 2.9%) [74], and in 25-month-old Nellore steers (2.65%) compared to Santa Gertrudis × Nellore crosses (3.64%), while the IMF content of Simmental × Nellore steers was intermediate and not different from either [105]. Two studies found no differences in IMF contents, both reporting low values (1.3–2.0% IMF) [66,78]. In one of these studies, the breeds were not reported [66], while in the other bulls of zebu, Holstein and European Brown Swiss purebreds and zebu crosses with Holstein, American Brown Swiss or European Brown Swiss were used. The lack of effect of genotype in the latter study was explained a result of the use of the combination of an anabolic implant and a β-agonist in the diet, which can significantly reduce the accumulation of fat in the muscle [122]. Lastly, one study reported a higher IMF content in *Bos indicus* than *Bos taurus cattle* (5.7% vs. 5.0%), but the breeds of cattle were not given. These IMF values are notably high compared to the other studies [68].

Finally, there are two reports on the impact of the feeding system on IMF content. Grain-finished bulls (*Bos indicus* and *Bos taurus*) produced meat with higher IMF contents than those finished on pasture (7.7% vs. 3.0%) [68]. Meat from steers (defined as “multiracial”) raised in feedlot had higher IMF content than those raised on pasture with supplementation (8.9% vs. 5.58%), both values being relatively high [98]. These findings are in accordance with the generally accepted conclusion that diets with a high content of concentrate cause rapid growth of cattle, and this is associated with a greater deposition of IMF [123].

### 4.2. Fatty Acids and Cholesterol

Beef IMF, regardless of where the cattle is raised, is comprised of over 20 individual fatty acids, of which six contribute more than 90% of the total fatty acid (TFA) content, myristic (C14:0), palmitic (C16:0), palmitoleic (C16:1), stearic (C18:0), oleic (18:1 cis-9) and linoleic (C18:2) acids [20]. Fatty acid profiles can vary with factors such as breed, feed and sex of the animal.

Given the decades of research on crossbreeding as a means to improve production traits and meat quality, including through increased fatness and marbling of otherwise lean *Bos indicus* cattle, it is not surprising that many of the studies on fatty acids focus on the impact of breed. In a Cameroonian study, while some differences were found, overall, genotype had a limited effect on the fatty acid profile of crossbred Simmental × Gudali and purebred Gudali bulls [106]. All of the animals were raised on pasture and were slaughtered at 20–41 months of age. These workers suggested that similar diets explained the lack of differences in the fatty acid profiles between breeds. Indeed, the levels of α-linolenic acid (C18:3n-3) and linoleic acid (C18:2n-6), both of dietary origin, were similar between the genotypes. High levels of PUFA (17.8% of TFA) were explained [106], not only by the effect of the relatively high proportion of phospholipids in muscle expected in the very lean animals but, also, by the high content of PUFA, particularly PUFA n-3, that characterizes fresh forage from pasture [124].

In another Cameroonian study using three local *Bos indicus* breeds, it was also found that breed had a limited effect on the fatty acid profile of meat from Gudali and Red Mbororo bulls raised on pasture and slaughtered at 3–5 years of age [125]. The only difference, albeit small, was a lower concentration of stearic acid (C18:0) in the Gudali bulls (18.0% vs. 19.8% of TFA). Gudali also had a lower concentration of stearic acid compared to White Fulani (20.7% of TFA). It was suggested that these differences could be a consequence of differences in gastrointestinal tract and rumen volume among breeds, which can influence the ruminal microbial ecosystem [68]. Ruminal biohydrogenation of dietary fat was concluded to have occurred to a lower extent in Gudali compared to in White Fulani cattle, given that, in addition to the aforementioned lower proportion of stearic acid in the IMF of Gudali, a higher proportion of α-linolenic acid (C18:3n-3; 2.34% vs. 1.61% of TFA) was found. Furthermore, docosapentaenoic acid (C22:5n-3), which is derived from α-linolenic acid, was also higher in Gudali than in White Fulani beef (1.62% vs. 1.17% of TFA). As already mentioned, α-linolenic acid is exclusively of dietary origin, and in addition, stearic acid is an end product of the biohydrogenation of dietary unsaturated fatty acids (UFA) [125].

Other differences in the fatty acid profiles of the Gudali and White Fulani were evident, while the Red Mbororo was generally not significantly different from either. Gudali bulls had higher tridecanoic acid (C13:0) (0.25% vs. 0.11% of TFA) and lower pentadecanoic acid (C15:0) (0.29% vs. 0.37% of TFA) relative to White Fulani. The pentadecanoic acid findings were explained by genetic differences between the breeds related to *de novo* C15:0 syntheses from propionate in adipose tissue [126]. Total SFA was lower and PUFA and n-3 PUFA were higher in Gudali compared to White Fulani. The SFA and MUFA were positively correlated with IMF and the PUFA was negatively correlated, suggested a consequence of the decrease in the phospholipids/neutral lipids ratio that arises from an increase in the IMF [127]. Reported PUFA/SFA ratios of 0.29 [125] and 0.33–0.36 [106] are lower than the minimum PUFA/SFA ratio of 0.45 recommended for human health [128]. The inability to achieve the recommended PUFA/SFA ratio is well-documented in both *Bos taurus* [129,130,131] and *Bos indicus* [68,107] cattle, a consequence of the extensive biohydrogenation of the dietary UFA by rumen microorganisms.

In another African study, further differences in the fatty acid profiles of two *Bos taurus* breeds (Borgou and Lagunaire), and Zebu Fulani were observed [58]. These cattle were raised on pasture and slaughtered at about 5 years of age. Zebu Fulani had higher contents of myristic (C14:0; 2.08% vs. 0.37% and 1.42% of TFA, respectively) and palmitic acids (C16:0; 22.4% vs. 14.5% and 19.3% of TFA, respectively) than Lagunaire and Borgou breeds. The content of α-linolenic acid varied from 2.46% to 3.81% of TFA but did not differ with breed. Zebu Fulani had the highest proportion of SFA (49.7% vs. 35.6% and 43.0% of TFA, respectively) and lowest total n-6 fatty acids (10.2% vs. 20.3% and 16.4% of TFA, respectively) when compared to Lagunaire and Borgou bulls. The ratio of PUFA/SFA fatty acids varied from 0.04 to 0.57 and was higher in Borgou than in Lagunaire.

In a Brazilian study, it was found that Nellore × Santa Gertrudis steers had higher SFA (508 vs. about 474 g/kg total fatty acid) and conjugated linoleic acid (CLA) isomer C18:2 cis-9, trans-11 (9.9 vs. 8.4 and 9.2 mg/g fat, respectively) and lower PUFA (46 vs. about 70 g/kg of TFA) than Nellore or Simmental × Nellore [105]. No differences in n-6 fatty acids were observed, but Simmental × Nellore cattle had lower n-3 than Nellore or Santa Gertrudis. In a Mexican study, it was found that fatty acid profiles of IMF from crossbreeds of 3/4 zebu or 3/4 European (based on Holstein crosses) differed, regardless of pasture or feedlot finishing [114]. The IMF from steers with *Bos indicus* dominance contained more myristic (31 vs. 26 mg/g fat), palmitic (255 vs. 238 mg/g fat), linoleic (64 vs. 38 mg/g fat) and linolenic acids (13 vs. 6 mg/g fat), but less stearic acid (191 vs. 226 mg/g fat) than steers with *Bos taurus* dominance.

While these few studies illustrate the complexity of research on the impact of breed on the fatty acid composition of beef, recent work demonstrates that it is even more complex than these studies would suggest. Indeed, a Brazilian study of pasture- and grain-finished purebred Nellore and crossbred Simmental × Nellore bulls found that, among the 43 individual fatty acids and indices of fatty acids in the IMF, 14 were affected by an interaction between the genetic group and the finishing system [117]. For the major groups of fatty acids, the interaction of the genetic group and the finishing system influenced the totals of the SFA and PUFA, while, for MUFA, only the effect of genetic group was significant. Of the 29 fatty acids and indices of fatty acids where the interaction was not significant, 11 were influenced by the genetic group and 25 by the finishing system. Overall, with only the exceptions of cholesterol, 18:1 trans-6,-7,-8, 18:1 trans-12 and the ratio 22:5n-3/18:3n-3, all the individual fatty acids and indices were affected by at least one of the factors considered or their interactions. These findings serve to illustrate that the importance of studying animals in their production environment cannot be overstated. The most prominent feature was the impact of the finishing system on the IMF and the fatty acid profile, but differences among the genetic groups were important. Differences among the genetic groups were minor with pasture finishing, but in grain finishing, the *Bos indicus* showed higher amounts of SFA and stearic acid and lower concentrations of fatty acids synthesized from linoleic and α-linolenic acids. Generally, animals finished on pasture produced meat with lower IMF, trans fatty acids and SFA contents and higher CLA and long-chain PUFA (20:5 n-3 and 22:5 n-3).

A number of studies have evaluated the impact of feed on fatty acid composition, and in particular, the manipulation of animal feed has been used as a method to improve the nutritional quality of meat. In general, pasture systems (not necessarily tropical) lead to an increase in PUFA in bovine meat compared to grain-based diets [132,133]. Diets rich in forage favor the growth of fibrolytic microorganisms responsible for the rumen production of CLA [134], and cattle fed forage have higher concentrations of linoleic, stearic, arachidonic (20:4n-6), eicosapentaenoic (20:5n-3) and docopentanaenoic (22:5n-3) acids in meat than those fed concentrates [135]. However, it must be kept in mind that pastures in climatically different environments can vary enormously. Furthermore, climatic variations in tropical regions can greatly impact pasture. For example, a Venezuelan study concluded that variations in linoleic acid and CLA contents in IMF could be explained by slaughter in two different seasons [136]. Brahman crosses were slaughtered at 17 and 19 months of age in April and June 1998, which coincided with the “El Niño” climatic effects, characterized by a prolonged dry season. A 24-month-old group was slaughtered between November and December 1998 under less severe environmental conditions. During drought periods, the fiber contents in plants in pastures increase. In addition, a higher quantity of soluble fiber creates an environment that promotes a greater production or a decreased utilization of CLA by rumen bacteria [137], explaining, at least in part, the higher CLA content in meat from the younger animals (1.76 mg/g IMF at 17 months, 1.98 mg/g IMF at 19 months and 1.20 mg/g IMF at 24 months). It should be noted that these workers also concluded that, considering the sparingly low lipid concentrations (˂2 g/100 g of fresh muscle), none of the meat could be considered a significant source of CLA. And, while one might think that feedlots would reduce variation among climatic regions given the global trade of grain, factors other than those related to climate may be significant. For example, it is reported that due to limitations of grain processing, the utilization of starch in feedlots in Brazil is not optimal, and levels fed are much lower than those used in North American feedlots [138]. Furthermore, there is a significant incorporation of forage and byproducts in the feeds of feedlot-raised beef.

In Mexico, a study found that steers of 3/4 zebu and 3/4 European cattle in a feedlot system consumed greater amounts of fatty acids compared to those on pasture (157 vs. 116 g/day SFA, 154 vs. 77 g/day MUFA and 189 vs. 135 g/day PUFA), but the latter consumed more alpha-linolenic acid [114]. Pasture-fed animals deposited lesser proportions of myristic and palmitic acids (32.4 and 255.4 mg/g fat, respectively) in the IMF than feedlot animals (21.8 and 236.5 mg/g fat, respectively), even though the consumption of these two fatty acids was similar with production system. No differences in the n-6/n-3 ratio (about 7.2) or total content of CLA were observed (14.4–16.8 mg/g fat). It was suggested that CLA is more likely located in the subcutaneous fat than in the IMF.

In another Mexican study, the concentration of the CLA isomer, C18:2 *cis*-9, *trans*-11, in beef from grazing cattle was slightly more than double that in meat from cattle-raised in a feedlot system [98]. In this experiment, steers defined as “multiracial” were raised on pasture to a final live weight of 320 kg or in feedlot to 480 kg. In the pasture-fed beef, concentrations of pentadecanoic (C15:0), heptadecanoic (C17:0) and stearic acids and CLA isomers (C18:2, n-6, C18:3, n-3, C18:2 *cis*-9 and *trans*-11) were higher, while beef from the feedlot system had higher concentrations of myristic, myristoleic (C14:1), oleic (C18:1 *cis*-9), elaidic (C18:1, n-9) and *cis*-10 heptadecanoic acids (C17:1*cis*-10).

In a Colombian study, meat from zebu cattle raised in four production systems (two silvopastoral systems, improved pasture and a traditional grazing system) was compared [139]. Myristic and palmitic acids were higher in meat from the traditional (3.65 and 32.6 g/100 g of TFA, respectively) than the improved pasture system (2.82 and 28.9 g/100 g of TFA, respectively), while linolenic acid was lower (0.96 vs. 2.30 g/100 g of TFA). The results from the silvopastoral systems differed, with one system showing a similar fatty acid profile to the traditional pasture system.

Aside from breed and feed, the potential of castration as a means to improve the meat quality in tropical beef is a research area that has piqued some interest. Higher levels of stearic acid were found in the IMF of meat from castrated Nellore cattle (around 21% of TFA) than from intact bulls (18.5% of TFA) in a Brazilian study comparing two methods of castration [107]. The linoleic acid concentration was lower in surgically castrated cattle than chemically castrated or intact bulls (3.5% vs. 4.5% and 5.1% of TFA, respectively). Regardless of the type of castration, the content of linolenic (about 0.9% vs. 1.8% of TFA), eicosapentaenoic (C20:5n-3; about 0.13% vs. 0.19% of TFA) and docosapentaenoic acids (C22:5n-3; 0.7–1.1% vs. 1.6% of TFA) was higher in IMF of meat from bulls compared to steers. The IMF from the surgically castrated animals contained less PUFA than that from chemically castrated cattle (8.2% vs. 9.7% of TFA), which was less than that from bulls (12.0% of TFA). This was explained by the fact that bulls exhibit a greater musculature development than steers [109], and PUFA is a major component of phospholipids in the cellular membranes of muscle tissues [140]. Steers also had lower SFA contents (49.0% and 52.2% of TFA for chemical and surgical castration, respectively) than bulls (47.3% of TFA), resulting in a smaller PUFA/SFA ratio of surgically castrated cattle than the bulls (0.16 vs. 0.25) and that from chemically castrated cattle being no different from either (0.20). The PUFA/SFA ratio increased as the fat content decreased, given that, at low levels of IMF, the contribution made by phospholipids is proportionately greater, and these are more unsaturated than the triacylglycerols, which themselves increased in proportion as the total lipid increased [141]. The n-6/n-3 ratios were higher in castrated animals (about 3.0) than intact bulls (2.1), but all were low.

Similar trends were observed in a Brazilian study of crossbred zebu x Aberdeen Angus on supplemented pasture [108]. The myristic (1.3% vs. 1.7% of TFA), linoleic (6.6% vs. 4.2% of TFA) and linolenic acids (1.2% vs. 0.6% of TFA) were all higher in bulls than steers. In addition, PUFA were higher in bulls (14.1% vs. 8.0% of TFA), resulting in a higher PUFA/SFA ratio than in steers (0.29 vs. 0.19). And, the n-6/n-3 ratios were lower in bulls than steers (2.4 vs. 3.0). Also observed were lower palmitic acid (23.5% vs. 25.0% of TFA) and MUFA (36% vs. 39% of TFA) in the IMF of bulls than steers.

The cholesterol content of IMF has also been measured in some studies. In a Venezuelan study, feed supplementation in pasture finishing had no impact on the cholesterol content (around 29 mg/100 g muscle tissue) of meat from Criollo Limonero steers slaughtered at 36 months of age after finishing on one of three systems: pasture, pasture supplemented with concentrate or pasture supplemented with legumes [112]. Another Venezuelan study found that cholesterol in meat from Brahman-influenced bulls and steers raised on pasture increased from 54.5 mg/100 g tissue at 19 months of age to 69.04 mg/100 g at 24 months of age [109]. A Brazilian study reported a lower cholesterol content in Nellore (46.6 mg/100 g muscle) and Nellore × Simmental steers (46.9 mg/100 g muscle) than in Nellore × Santa Gertrudis steers (48.3 mg/100 g muscle) [105].

Finally, it must be noted that there are a number of studies aimed at identifying the means to genetically select traits in tropically adapted cattle, including a couple on fatty acid composition. Some workers have suggested that the demonstrated existence of genetic variation in the IMF from feedlot-finished Nellore steers allows the possibility to increase the proportion of healthy and favorable beef fatty acids through selection [142]. Others have found that the selection for decreased subcutaneous fat resulted in decreased proportions of oleic acid with concomitant increases in stearic, myristic and palmitic acids [143]. It was suggested that selection for decreased fat at a given weight will result in a decrease in the proportion of MUFA in the subcutaneous fat in the carcass, with a corresponding increase in the proportions of less healthy SFA.

### 4.3. Minerals

In a recent review on minerals in meat, particularly from animals raised in tropical regions, Ribeiro, Mourato and Almeida [144] described how concentrations are influenced by a vast array of factors, including species, sex, genotype, production stage, region, climate, tissue characteristics and animal management practices, namely rearing systems and nutrition. However, there are few studies on mineral contents of meat from beef raised in tropical environments.

Investigating the use of trace elements as fingerprints for traceability of Brazilian beef, mineral content was analyzed in meat from cattle raised in five different environments, of which three were tropical [145]. While relatively few differences were observed, these differences were sufficient to conclude that chemical traceability of Brazilian beef according to the biome of origin was feasible. Differences in mineral contents of the muscle tissues included the largest mass fractions of bromine and selenium in the Amazônia biome (tropical rainforest) and the lowest mass fraction of zinc in the Pantanal biome (tropical savanna).

In Venezuela, no differences in mineral contents of meat from Criollo Limonero steers fattened on three different feed systems were observed [112,113], nor was any difference found in mineral content with carcass grade of bulls, steers and heifers obtained from a commercial slaughterhouse in Venezuela [146]. In another study on carcass grade of Venezuelan beef, subprimal cuts were obtained from graded carcasses [115,116]. Again, mineral content did not vary with the carcass grade, but did show lower concentrations of Ca, Fe and Zn and higher P and K than meat imported from the US.

Also in Venezuela, no impact of sex on mineral content was observed in meat from zebu-influenced steers and bulls slaughtered at 17, 19 and 24 months [147]. However, an age effect was found for all of the minerals studied. As animal age increased from 17 to 24 months, concentrations of Na (59.5 vs. 71.2 mg/100 g fresh tissue), K (321 vs. 400 mg/100 g fresh tissue), Ca (3.54 vs. 8.17 mg/100 g fresh tissue), Mg (21.8 vs. 23.6 mg/100 g fresh tissue) and Zn (3.66 vs. 3.83 mg/100 g fresh tissue) increased, while those of P (203 vs. 195 mg/100 g fresh tissue), Mn (0.02 vs. 0.01 mg/100 g fresh tissue), Fe (2.34 vs. 1.75 mg/100 g fresh tissue) and Cu (0.16 vs. 0.09 mg/100 g fresh tissue) decreased. When the values at 19 months of age were included, it was noted that variations in mineral contents did not always show an apparent trend. While statistical differences were not identified, Na, P, Mn, Fe and Cu contents were similar at 17 and 19 months of age, and K and Zn were intermediary between 17 and 24 months, but Ca and Mg were the highest at 19 months. It was suggested that an effect of feed (produced by a drought period) could have impacted the findings. The amount and quality of grasses were reduced for the animals slaughtered at 17 and 19 months of age, while the slaughter of the 24-month-old animals occurred under less severe environmental conditions, a phenomenon that could have produced changes in the intramuscular accumulation of some minerals.

Indeed, Ribeiro, Mourato and Almeida [144] describe how the impact of climatic conditions on the soil and pasture is a determinant factor for the mineral concentrations of edible tissues. In tropical countries, pastures suffer a seasonal effect on their quantities and qualities, and fluctuations of feed quality may cause mineral imbalances throughout the year, with dry season being critical due to the lignification of natural pastures and water/feed scarcity. In addition, in the wet season, heavy rains may cause nutrient leaching and, consequently, reduce the mineral contents of forage and the availability to grazing ruminants [144].

## 5. Conclusions

Research on tropical beef quality and composition is reported from a limited number of countries, a consequence of a lack of access to research funding, resources and infrastructure. Studies that are reported have often been based on a piecemeal approach, using limited numbers of animals and short durations. Regardless, consolidating the findings from these studies has allowed the demonstration of an axiomatic basis defining “tropical beef” as a concept.

Tropical beef is the meat obtained from cattle raised in tropical environments. The majority of the tropical cattle population remains largely uncharacterized, and production systems in the Tropics are diverse, but converge on the use of indigenous and *Bos indicus* breeds or *Bos indicus*-influenced crossbreeds under pasture feeding regimes. No one gender is used throughout tropical production systems, and while some systems allow cattle to be slaughtered at ≤2 years of age, generally, animals are ≥3 years at slaughter. These production systems generally produce lean, low-yielding carcasses and tough (>46 N), lean (≤3.6% IMF) meat, with a macronutrient composition otherwise similar to beef from animals raised elsewhere (72–74% moisture and 20–24% protein). Fatty acid profiles depend on the breed and production systems, while mineral content is influenced by the environment. Although lean and tough, tropical beef is highly acceptable to the consumers it serves and is culturally and traditionally relevant. In many countries, tropical cattle have important functions, ranging from the provision of food and income to socio-economic and cultural roles. Indeed, tropical cattle contribute to food security and income in developing regions, particularly for smallholder beef farmers for whom producing meat in a sustainable manner is an important challenge.

## Figures and Tables

**Table 1:** Research on the impact of production factors, quality grade and aging time (treatments under study in bold) on the Warner Bratzler shear force (or equivalent) in meat from beef raised in tropical environments ^1^.

Country	Breed or Purchased Meat	*n*	Sex	Age (Months)	Feed	Other	Shear Force (N)	Days p.m. ^2^	Muscle ^3^	Ref
*No Comparison*										
Brazil	Nellore	1329	Bulls	21–24	Feedlot-finished		59	2	LT	[75]
							58	7		
							62	9		
Mexico ^4^	Purchased steaks	20					41		LD	[76]
Puerto Rico	Non-specified “typical” breeds	105	Male, female	≤30, ≥36	Pasture		46	1	LL	[77]
							43		St	
							49		Sm	
										
*Breed*										
Brazil	**Nellore**	306	Steers, heifers	22–24	Feedlot-finished		42 ^a^	15	LD	[70]
	**Nellore × Angus**						33 ^b^			
	**Brahman × Nellore**						39 ^ab^			
Brazil	***Bos taurus***	160	Bulls	26–40	Pasture or concentrate-finished		74	1	LT	[68]
	***Bos indicus***						88	1		
	***Bos taurus***						54	10		
	***Bos indicus***						60	10		
Brazil	**Guzerat × Holstein**	36	Bulls	26	Feedlot-finished		43 ^a^	1	LL	[69]
	**Guzerat × Nellore**						51 ^b^			
	**½Simmental × ¼Guzerat × ¼Nellore**						50 ^b^			
Australia	**Brahman × Shorthorn**	170	Bulls	36–84	Pasture		75	1	LD	[60]
	**Shorthorn**						75			
Australia	**Brahman × Shorthorn**	240	Cows	60–120	Pasture		89	1	LD	[60]
	**Shorthorn**						96			
Australia	**Brahman**	50	Steers	About 22	Feedlot-finished		39 ^a^	15	LD	[64]
	**Brahman × Senepol**						34 ^b^			
Mexico	**Brahman**	20	Bulls	18–24	Feedlot	β-adrenergic agonist used	54 ^a^	2		[74]
	**Charolais**						49 ^b^			
Mexico	**Zebu**	90	Bulls		Concentrate + forage finishing	β-adrenergic agonist used, acetate + estradiol implants	80 ^a^	2	LD	[78]
	**Zebu × Holstein**						62 ^b^			
	**Zebu × American Brown Swiss**						63 ^b^			
	**Zebu × European Brown Swiss**						63 ^b^			
	**Holstein**						61 ^b^			
	**European Brown Swiss**						68 ^b^			
Benin	**Zebu Fulani**	25	Bulls	60	Pasture		102	1	LT	[58]
	**Lagunaire**						91	1		
	**Borgou**						122	1		
	**Zebu Fulani**						60	3		
	**Lagunaire**						51	3		
	**Borgou**						90	3		
	**Zebu Fulani**						38	9		
	**Lagunaire**						37	9		
	**Borgou**						66	9		
Cameroon	**Goudali**	60	Bulls	36–60	Pasture		112 ^b^	8	LT	[56]
	**White Fulani**						72 ^b^			
	**Red Mbororo**						78 ^b^			
Venezuela	**Brahman,**	71	Bulls		Pasture-finished, Supplement-finished		40 ^ab^	2	LD	[79]
	**Brahman × Angus,**						36 ^c^			
	**Brahman × Gelbvieh**						38 ^bc^			
	**Brahman × Limousin,**						36 ^bc^			
	**Brahman × Romosinuano**						35 ^c^			
	**Brahman × ¾ *Bos taurus***						43 ^a^			
									
*Sex*										
Brazil	Nellore, Nellore × Angus, Brahman × Nellore	306	**Steers**	22–24	Feedlot-finished		39	15	LD	[70]
			**Heifers**				36			
Brazil	Angus × (Limousin × Nellore), Angus × (Simmental × Nellore)	225	**Bulls**	12.5, 18	Pasture-finished, feedlot-finished		84 ^a^	1	LD	[80]
			**Heifers**				94 ^b^			
Costa Rica	¾Brahman × ¼Charolais	47	**Steers-3**	61–66	Pasture		86 ^e^	2, 7, 14, 28	LL	[73]
			**Steers-7**				93 ^f^		LL	
			**Steers-12**				91 ^ef^		LL	
			**Bulls**				100 ^g^		LL	
			**Steers-3**				64 ^bc^		GM	
			**Steers-7**				66 ^bcd^		GM	
			**Steers-12**				70 ^cd^		GM	
			**Bulls**				73 ^d^		GM	
			**Steers-3**				61 ^b^		St	
			**Steers-7**				63 ^bc^		St	
			**Steers-12**				63 ^bc^		St	
			**Bulls**				61 ^b^		St	
			**Steers-3**				40 ^a^		PM	
			**Steers-7**				39 ^a^		PM	
			**Steers-12**				38 ^a^		PM	
			**Bulls**				39 ^a^		PM	
										
*Age or Dentition (Permanent Incisors)*										
Australia ^6^		198	Cows	**2 teeth**	Pasture		99	1	LD	[61]
				**4 teeth**			97			
				**6 teeth**			82			
				**8 teeth**			96			
Australia ^6^		168	Steers	**4 teeth**	Pasture		82	1	LD	[61]
				**6 teeth**			77			
				**8 teeth**			76			
Brazil	Nellore	60	Steers	**20–24**			68	15	LD	[81]
				**30–36**			68			
				**42–48**			57			
Puerto Rico	Non-specified “typical” breeds	105	Male, female	**≤30,**	Pasture		30 ^a^	1	LL, St, Sm	[77]
				**≥36**			46 ^b^			
										
*Feed*										
Brazil	*Bos indicus, Bos Taurus*	160	Bulls	26–40	**Pasture-finished**		85	1	LT	[68]
					**Concentrate-finished**		77	1		
					**Pasture-finished**		59	10		
					**Concentrate-finished**		55	10		
Brazil	Nellore	30	Bulls	22	**No glycerin in dry feed**		47	1	LD	[82]
					**7.5% glycerin**		46			
					**15% glycerin**		37			
					**22.5% glycerin**		44			
					**30% glycerin**		40			
Brazil	Nellore	60	Bulls	22	**No glycerin in dry feed**		30	1	LD	[83]
					**Glycerin + corn**		32			
					**Glycerin + soybean hulls**		28			
Australia	Brahman, Brahman × (Brahman x Santa Gertrudis, sanga × Belmont Red, Angus, Hereford, Shorthorn, Charolais, or Limousin)	349	Heifers, Steers	22–24	**Pasture-finished (heifers)**		55		LD	[63]
					**Feedlot-finished (heifers)**		48			
					**Feedlot-finished (steers)**		47			
Venezuela	Brahman, Brahman × (Gelbvieh, Romosinuano, Limousin, Angus or ¾ *Bos Taurus*)	71	Bulls		**Pasture-finished**		58 ^a^	2	LD	[79]
					**Supplement-finished**		67 ^b^			
										
*Other: Fat Class (F), Ageing Time of the Meat*										
Brazil	Nellore	60	Steers	22–48		**F: Slight**	70 ^a^	15	LD	[81]
						**F: Average**	59 ^b^			
Costa Rica	¾Brahman × ¼Charolais	47	Steers castrated at 3, 7 or 12 months, bulls	61–66	Pasture		102 ^i^	**2**	LL	[73]
							96 ^h^	**7**	LL	
							96 ^h^	**14**	LL	
							76 ^f^	**28**	LL	
							83 ^g^	**2**	GM	
							68 ^e^	**7**	GM	
							64 ^de^	**14**	GM	
							57 ^c^	**28**	GM	
							66 ^e^	**2**	St	
							61 ^cd^	**7**	St	
							62 ^cd^	**14**	St	
							60 ^cd^	**28**	St	
							44 ^b^	**2**	PM	
							38 ^ab^	**7**	PM	
							38 ^ab^	**14**	PM	
							36 ^a^	**28**	PM	

^1^ Shear force data in a given study with differing superscripts (down a column) are significantly different (*p* ≤ 0.05). ^2^ Number of days postmortem (p.m.) at which the shear force measure was undertaken. ^3^ *Longissimus dorsi* (LD), *Longissimus thoracis* (LT), *Longissimus lumborum* (LL), *Semimembranosus* (Sm), *Semitendinosus* (St), *Gluteus medius* (GM) and *Psoas major* (PM). ^4^ Veracruz data only. ^5^ Castrated at 3 months (Steers-3), 7 months (Steers-7) or 12 months (Steers-12). ^6^ Northern Queensland data only.

**Table 2:** Research on the impact of production factors, quality grade or muscle (treatments under study in bold) on the moisture, fat and protein in meat from beef raised in tropical environments.

Country	Breed or Purchased Meat	*n*	Sex	Age (Months)	Feed	Other	Moisture (%)	Protein (%)	IMF (%)	Ref
*No Comparison*										
Venezuela	Brahman × (Brahman, Black Angus, Red Angus, Romosinuano or Charolais)	17	Bulls, steers	24	Pasture		75.3	20.9		[99]
Venezuela	Zebu crossbreeds, dairy crossbreeds	145	Bulls, steers, heifers	30–48	Pasture		73.9			[100,101]
Venezuela	Brahman, Zebu × Brown Swiss, *Bos taurus* (Angus, Limousin, Gelbvieh, Criollo Romosinuano) × Brahman	20	Bulls, steers	30–60	Pasture		73.0	22.3	2.6	[102]
Venezuela	Purchased meat	20					74.2	22.4	3.6	[103]
Mexico ^2^	Purchased steaks	20					73.5	19.4	1.9	[76]
Mexico ^3^	Purchased steaks	20					72.2	22.3	3.6	[92]
Indonesia	Simmental × Ongole						72.4	21.8	3.5	[104]
										
*Breed*										
Brazil	**Nellore**	45	Bulls	24	Feedlot-finished		72.2	25.1 ^a^	1.7	[66]
	**½Nellore × ½European**						73.2	23.8 ^b^	2.0	
	**¼Nellore × ¾European**						73.5	23.7 ^b^	1.8	
Brazil	***Bos taurus***	160	Bulls	26–40	Pasture- or concentrate-finished		73.3	19.7	5.0 ^a^	[68]
	***Bos indicus***						72.7	19.8	5.7 ^b^	
Brazil	**Nellore**	18	Steers	25	Pasture-finished		74.1 ^a^	23.4 ^a^	2.7 ^a^	[105]
	**Simmental × Nellore**						73.9 ^ab^	23.0 ^ab^	3.1 ^a^	
	**Santa Gertrudis × Nellore**						73.3 ^b^	22.7 ^b^	3.6 ^b^	
Cameroon	**Goudali**	50	Bulls	20–41	Pasture		76.6	20.1	0.60	[106]
	**Italian Simmental × Goudali**						76.0	20.5	0.76	
Cameroon	**Goudali**	60	Bulls	36–60	Pasture		74.9	22.1 ^a^	1.1	[56]
	**White Fulani**						75.5	21.5 ^b^	1.4	
	**Red Mbororo**						75.8	21.6 ^b^	0.9	
Mexico	**Zebu**	90	Bulls		Concentrate + forage-finished	β-adrenergic agonist used; trenbolone acetate + estradiol implants	74.1	20.3	1.7	[78]
	**Zebu × Holstein**						74.3	20.8	1.7	
	**Zebu × American Brown Swiss**						74.4	20.6	1.7	
	**Zebu × European Brown Swiss**						74.0	20.3	2.0	
	**Holstein**						75.2	19.8	1.6	
	**European Brown Swiss**						75.4	20.8	1.3	
Mexico	**Brahman**	20	Bulls	18–24	Feedlot	β-adrenergic agonist used	73.7 ^a^		2.9 ^a^	[74]
	**Charolais**						75.1 ^b^		2.4 ^b^	
Benin	**Zebu Fulani**	25	Bulls	60	Pasture			21.7	1.25	[58]
	**Lagunaire**							19.5	0.44	
	**Borgou**							20.7	0.61	
										
*Sex*										
Brazil	Nellore crosses	30	**Steers Imm ^4^**	36	Pasture + supplement-finished		75.7	21.1	1.6 ^a^	[107]
			**Steers Surg**				74.7	20.9	2.2 ^b^	
			**Bulls**				76.2	19.9	1.2 ^c^	
Brazil	Zebu × Aberdeen Angus	27	**Steers**	27	Pasture + supplement-finished		73.1 ^a^	23.8 ^a^	2.0 ^a^	[108]
			**Bulls**				76.2 ^b^	22.9 ^b^	1.0 ^b^	
Venezuela	Brahman	34	**Steers**	19, 24	Pasture				2.1 ^a^	[109]
			**Bulls**						1.8 ^b^	
										
*Age*										
Brazil	Nellore	60	Steers	**20–24**			72.3 ^a^		4.2 ^a^	[67]
				**30–36**			71.9 ^ab^		5.0 ^ab^	
				**42–48**			71.0 ^b^		5.7 ^b^	
India	Kangayam	12		**12–18**			76.1 ^a^	20.7 ^a^	2.1 ^a^	[110]
				**>36**			74.0 ^b^	22.0 ^b^	2.9 ^b^	
Puerto Rico	Non-specified “typical” breeds	105	Male, female	**≤30**	Pasture		74.6	20.1	1.9 ^a^	[77]
				**≥36**			73.8	20.6	2.7 ^b^	
Venezuela	Brahman	34	Bulls, steers	**19**	Pasture				1.4	[109]
				**24**					2.0	
										
*Feed*										
Brazil	*Bos indicus, Bos Taurus*	160	Bulls	26–40	**Pasture-finished**		73.8 ^a^	21.4 ^a^	3.0 ^a^	[68]
					**Concentrate-finished**		72.2 ^b^	18.2 ^b^	7.7 ^b^	
Brazil	Nellore	60	Bulls	22	**No glycerin in dry feed**				2.5	[83]
					**Glycerin + corn**				3.0	
					**Glycerin + soybean hulls**				2.9	
Brazil	Nellore	30	Bulls	22	**No glycerin in dry feed**		76.3	21.8	2.1	[82]
					**7.5% glycerin**		75.1	22.1	2.5	
					**15% glycerin**		76.0	21.2	2.3	
					**22.5% glycerin**		75.	22.4	2.3	
					**30% glycerin**		75.8	21.8	1.9	
Venezuela	Brahman, Angus, Romosinuano, Senepol, Simmental, commercial zebu crosses	89	Bulls, steers		**Pasture**	Implants (Ralgro, Revalor)	73.9 ^a^	21.4		[96,111]
					**Pasture + supplement**		74.2 ^b^	21.7		
Venezuela	Criollo Limonero	23	Steers	36	**Pasture**	-	71.9	22.4	2.9	[112,113]
					**Pasture + concentrate**		71.5	22.9	3.1	
					**Pasture + legume**		72.2	22.3	3.1	
Mexico	“Multi-racial”	80	Steers	22–38	**Pasture + supplement**		71.6 ^a^	21.3	5.6 ^a^	[98]
					**Feedlot**		67.3 ^b^	22.7	8.9 ^b^	
Mexico	¾ Zebu, ¾ *Bos taurus* (Holstein crosses)	52	Steers		**Pasture-finished**		71.3	20.8	2.3	[114]
					**Feedlot-finished**		73.8	22.2	2.2	
										
*Other: Fat Class (F), Carcass Grade (C), Muscle (M), Implants (I)*										
Brazil	Nellore	60	Steers			**F: Slight**	72.3 ^a^		4.2 ^a^	[67]
						**F: Medium**	71.1 ^b^		5.7 ^b^	
Venezuela	Angus, ¾ Brahman (*n* = 18); purchased meat (*n* = 40)	58	Steers		Pasture + supplement-finished	**C ^5^: BF A**	74.4	20.5	3.5	[115,116]
						**C: BF AA**	74.3	20.4	4.0	
						**C: LD A**	74.7	21.9	2.0	
						**C: LD AA**	74.0	21.5	3.0	
Venezuela	Brahman, Angus, Romosinuano, Senepol, Simmental, commercial zebu crosses	77	Bulls		Pasture ± supplement	**I ^6^: Ral-Ral**	60.0	35.5	3.6 ^a^	[96]
						**I: Rev-Ral**	59.4	35.9		
Venezuela	Brahman, Angus, Romosinuano, Senepol, Simmental, commercial zebu crosses	89	Bulls, steers		Pasture ± supplement	**I ^7^: Ral-Ral**			1.3 ^a^	[111]
						**I: Rev-Ral**			1.4 ^b^	
										
*Breed × Production System Interaction*										
Brazil	**Nellore**	134	Bulls	23.5, 27.5	**Feedlot-finished**				2.7 ^a^	[117]
	**Simmental × Nellore**				**Feedlot-finished**				2.1 ^b^	
	**Nellore**				**Pasture-finished**				1.3 ^c^	
	**Simmental × Nellore**				**Pasture-finished**				1.6 ^c^	

^1^ Moisture, protein or fat content means in a given study with differing superscripts (down a column) are significantly different (*p* ≤ 0.05). ^2^ Veracruz data only. ^3^ Villahermosa data only. ^4^ Chemical (Imm) or surgical (Surg) castration. ^5^ *Biceps femoris* (BF) or *longissimus dorsi* (LD) and Venezuelan carcass grade AA or A. ^6^ Ralgro (Ral) implants consisting of zeranol, an anabolic agent; Revalor (Rev) implants consisting of the anabolic steroid trenbolone acetate and the estrogen hormone, estradiol.
